# Molecular Basis of High-Blood-Pressure-Enhanced and High-Fever-Temperature-Weakened Receptor-Binding Domain/Peptidase Domain Binding: A Molecular Dynamics Simulation Study

**DOI:** 10.3390/ijms26073250

**Published:** 2025-03-31

**Authors:** Xubin Xie, Yu Zhang, Ying Fang, Jianhua Wu, Quhuan Li

**Affiliations:** Institute of Biomechanics, School of Biology and Biological Engineering, South China University of Technology, Guangzhou Higher Education Mega Centre, Panyu District, Guangzhou 510006, China; xiexubin@163.com (X.X.); zhangyu970724@163.com (Y.Z.); yfang@scut.edu.cn (Y.F.)

**Keywords:** COVID-19, RBD, ACE2, hypertension, high temperature, molecular dynamics simulations

## Abstract

The entry and infection of the Severe Acute Respiratory Syndrome Coronavirus 2 virus (SARS-CoV-2) involve recognition and binding of the receptor-binding domain (RBD) of the virus surface spike protein to the peptidase domain (PD) of the host cellular Angiotensin-Converting Enzyme-2 (ACE2) receptor. ACE2 is also involved in normal blood pressure control. An association between hypertension and COVID-19 severity and fatality is evident, but how hypertension predisposes patients diagnosed with COVID-19 to unfavorable outcomes remains unclear. High temperature early during SARS-CoV-2 infection impairs binding to human cells and retards viral progression. Low body temperature can prelude poor prognosis. In this study, all-atom molecular dynamics simulations were performed to examine the effects of high pressure and temperature on RBD/PD binding. A high blood pressure of 940 mmHg enhanced RBD/PD binding. A high temperature above 315 K significantly weakened RBD/PD binding, while a low temperature of 305 K enhanced binding. The curvature of the PD α1-helix and proximity of the PD β3β4-hairpin tip to the RBM motif affected the compactness of the binding interface and, hence, binding affinity. These findings provide novel insights into the underlying mechanisms by which hypertension predisposes patients to unfavorable outcomes in COVID-19 and how an initial high temperature retards viral progression.

## 1. Introduction

Coronavirus disease 2019 (COVID-19) caused by Severe Acute Respiratory Syndrome Coronavirus 2 (SARS-CoV-2) emerged in December 2019 and rapidly spread worldwide. This pandemic posed an unprecedented health crisis [1,2,3]. SARS-CoV-2 is an enveloped, single-stranded RNA virus belonging to the genus Betacoronavirus [2,3]. The mechanisms of SARS-CoV-2 transmission and pathogenesis involve the initial recognition and binding of the viral surface spike protein to the Angiotensin-Converting Enzyme 2 (ACE2) receptor of the host cell, followed by conformational changes in the spike protein that facilitate the formation of the fusion machinery.

**Figure 1:**
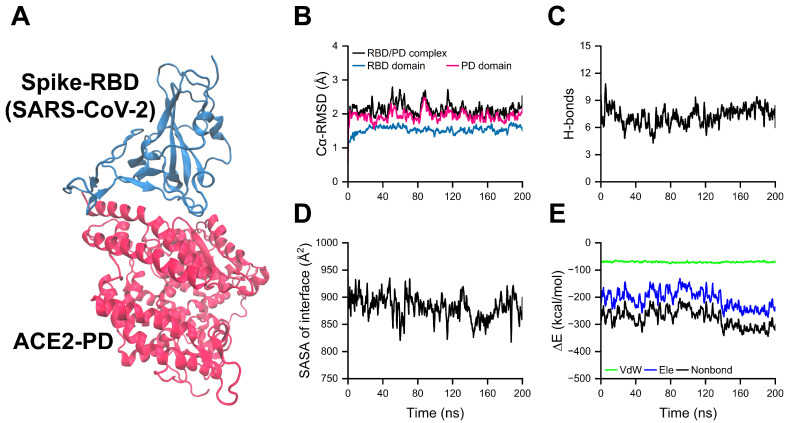
Equilibration of the RBD/PD complex. (**A**) Schematic diagram of the spike-RBD/ACE2-PD complex used for equilibration, where the RBD from the SARS-CoV-2 spike protein and the PD from the human ACE2 receptor are depicted in NewCartoon style and colored dark cyan and red, respectively. (**B**) Time courses of the Cα-RMSD values for the RBD/PD complex, RBD, and PD during equilibration, with their respective initial coordinates serving as references. (**C**–**E**) Time courses of the number of hydrogen bonds (H-bonds), solvent-accessible surface area (SASA) of the binding interface, and binding energy (∆E) during equilibration. ∆E includes electrostatic energy (Ele) and van der Waals energy (VdW), and the sum of them is referred to as nonbonded interactions (Nonbond).

These processes are regulated by two subunits of the spike protein, S1 and S2, which mediate receptor binding and membrane fusion, respectively [4,5,6,7]. The S1 subunit consists of an N-terminal domain (NTD), a receptor-binding domain (RBD), and subdomains 1 and 2 (SD1 and SD2). The RBD is primarily responsible for ACE2 recognition [8,9]. The RBD has a twisted five-stranded antiparallel β sheet with short connecting helices and loops that form the core. Outside the core region, there is an extended insertion termed the RBM motif, which contains short β5 and β6 strands, α4 and α5 helices, and loops (mostly disordered), which directly contact the N-terminal peptidase domain (PD) of ACE2 (Figure 1).

ACE2 is a type I transmembrane protein with two major functions. One function is as an endogenous counter-regulator of the Renin–Angiotensin–Aldosterone System (RAAS) [10,11]. The other is as a host cell receptor for SARS-CoV [12,13]. ACE2 was recently confirmed as the host cell receptor for SARS-CoV-2 [8,9,14], establishing a direct link between COVID-19 and the RAAS. In the RAAS [10,11], ACE induces vasoconstriction and increases blood pressure through the ACE/Angiotensin II (Ang II)/Angiotensin type 1 receptor (AT1R) axis, whereas ACE2 counteracts the action of ACE, induces vasodilation, and decreases blood pressure through the ACE2/Ang-(1-7)/AT2R axis. These two axes are coexpressed in tissues, such as the lungs, heart, kidney, and vasculature, and the balance between them is important for maintaining normal physiological functions, including blood pressure control.

Hypertension, also known as high blood pressure, is a serious public health problem affecting approximately 31.1% of adults worldwide. It is characterized by persistently elevated blood vessel pressure [15,16]. Since the onset of the COVID-19 pandemic, in-hospital mortality has been linked to the presence of several comorbidities, with hypertension being the most prevalent [17,18,19]. A plethora of evidence indicates that hypertension is associated with a higher susceptibility to SARS-CoV-2 infection and increased severity and fatality of COVID-19 [16,20,21,22,23,24,25,26]. The coexistence of hypertension and SARS-CoV-2 infection can be a double blow for patients. However, the precise mechanism by which hypertension predisposes patients to unfavorable COVID-19 outcomes remains unclear. A lack of direct evidence exists regarding whether and how hypertensive conditions affect RBD/PD binding, especially considering that the binding of the spike protein to human ACE2 initiates SARS-CoV-2 entry.

Blood pressure is created by the force of blood pushing against the walls of blood vessels (arteries) as it is pumped by the heart. However, hypertension is not merely a consequence of high blood pressure but also the combined effects of various factors, including cardiac output, vascular resistance, and vascular wall elasticity [27,28]. These factors collectively contribute to the complex pathophysiology of hypertension. Hu et al. reported that the tensile force generated by the bending of the host cell membrane enhances spike protein recognition by ACE2 [29]. Given the widespread expression and distribution of ACE2 in the cardiovascular system, changes in blood pressure may alter the mechanical environment surrounding ACE2. Therefore, the first hypothesis in this study is that elevated blood pressure in hypertensive patients may enhance the binding affinity between spike-RBD and ACE2-PD, leading to increased viral attachment to human cells, accelerated viral progression, increased viral loads, and prolonged viral shedding.

Fever is an evolutionarily conserved and beneficial physiological response and has been identified as a common initial symptom of SARS-CoV-2 infection [3,30,31,32,33,34]. The magnitude of temperature elevation may serve as an indicator of inflammation severity [35]. Although a prolonged fever is generally clinically undesirable, a mild and reversible fever can enhance the immune response against pathogens [36]. High temperatures during the early stages of SARS-CoV-2 infection impair viral binding to human cells, slowing infection progression [37]. Conversely, a lower body temperature at initial presentation serves as an indicator of poor prognosis, as patients with a body temperature ≤ 36 °C exhibited significantly higher mortality rates compared to normothermic patients [35,38]. Recent in vitro and in silico studies have shown that temperature influences the binding affinity between spike proteins and human ACE2 [37,39,40,41,42]. Therefore, the second hypothesis in this study is that high temperatures may weaken the binding of spike-RBD and ACE2-PD, resulting in reduced viral attachment to human cells, slower viral progression, and lower viral load. Consequently, this could delay multiorgan damage and provide more time for the immune system to eliminate the virus before severe organ failure occurs. Similarly, fever is not merely an elevation of temperature; it is instead a manifestation of the complicated inflammatory responses triggered by viral invasion [34]. To test these two hypotheses, we primarily focused on the initial step of SARS-CoV-2 infection to investigate the interaction between the spike protein and the human ACE2 receptor. After that, the spike-RBD/hACE2-PD complex was further studied. Here, we assume that hypertensive patients with higher blood pressures expose the virus to elevated blood pressures during viral infection and that the key binding unit, the RBD/PD complex, is subjected to higher pressures. Similarly, the RBD/PD complex is subjected to high temperatures in patients with fevers. We simulated the effects of elevated blood pressure and fever temperature on the binding of the RBD/PD complex by maintaining constant system pressure (e.g., hypertension level of 940 mmHg) and temperature (e.g., the upper limit of fever of 315 K) during all-atom molecular dynamics (MD) simulations, using the crystal structure of the RBD/PD complex as the initial conformation [8]. Our results demonstrated that high blood pressure (940 mmHg) and low temperature (305 K) significantly enhanced RBD/PD binding, whereas temperatures exceeding 315 K markedly weakened this interaction. The curvature of the α1-helix from the PD and the proximity of the tip of the β3β4-hairpin from the PD to the RBM motif influenced the compactness of the binding interface, thereby affecting binding affinity. These findings are expected to provide novel insights into the mechanisms by which hypertension predisposes patients to unfavorable outcomes in COVID-19 and how initial high temperatures may retard viral progression.

## 2. Results

### 2.1. Equilibration of RBD/PD Complexes

To obtain a stable conformation of the RBD/PD complex under physiological conditions, equilibration under the isobaric–isothermal ensemble (NPT; 1 atm and 310 K) was first performed for 200 ns using the crystal structure of the complex (Protein Data Bank ID: 6M0J) [8] as the starting conformation (Figure 1A). Time courses of the root mean square deviation of Cα atoms (Cα-RMSD) showed that the RBD, PD, and RBD/PD complex reached plateaus after ~25, ~50, and ~50 ns, respectively. The Cα-RMSD of the complex followed nearly the same pattern as that of the PD, indicating that the RBD/PD complex as a whole reached its stable state and was mainly governed by the PD, which covers approximately 75% of the residues in the entire complex (Figure 1B). The time courses of the number of hydrogen bonds formed on the binding interface of the complex (H-bonds), the solvent-accessible surface area (SASA) of the binding interface, and binding energy (∆E) showed that a stable binding interface of the whole complex formed after the conformation adjustment in the first ~90 ns (Figure 1C–E). The time courses of H-bonds formed at the binding interface of the complex, the SASA of an interface, and binding energy (∆E, calculated in vacuum) indicated that an initial conformational adjustment occurred within the first ~90 ns. The average values of H-bonds, SASA, and ∆E stabilized at 7.25 ± 1.95, 877.88 ± 34.19 Å^2^, and −278.88 ± 40.81 kcal/mol, respectively (Figure 1C–E). These results indicate the formation of a stable binding interface concurrent with the stabilization of the entire complex. Conformations from the equilibrated stage were randomly selected as starting conformations for subsequent simulations to investigate the effects of high pressure and temperature on RBD/PD binding.

**Figure 2:**
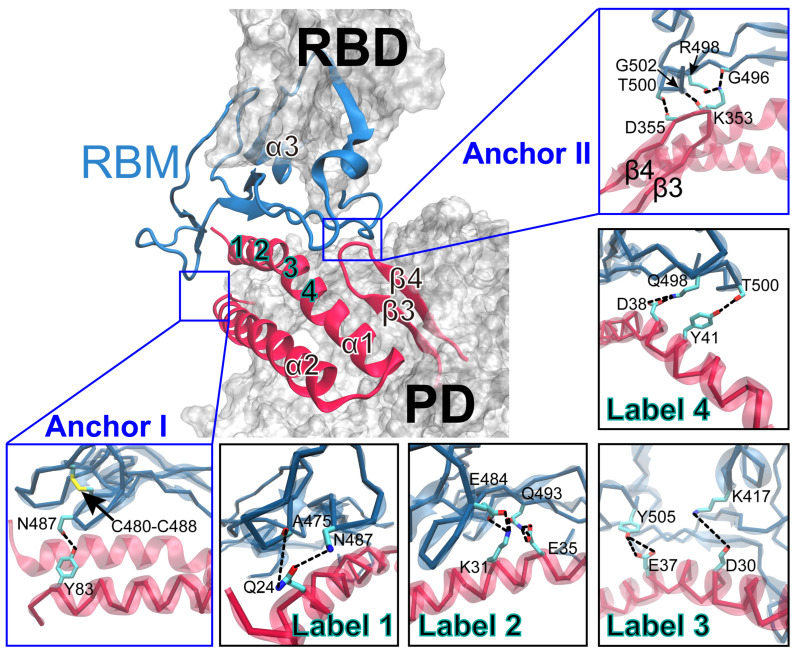
Binding interface of the equilibrated RBD/PD complex. The overall structure of the RBD/PD complex is illustrated on the upper left, in which the secondary structures forming the binding interface are depicted in NewCartoon representation, while the remaining region is shown in white Surf. The rectangular boxes on the right and bottom provide enlarged views that highlight the key H-bonds formed at the binding interface. The involved residues are depicted in licorice, and the H-bonds are shown as black dotted lines. The key structures in the RBD (the RBM motif and α3-helix) and the PD (α1-helix, α2-helix, β3-strand, and β4-strand) are colored dark cyan and red, respectively. The H-bonds in Anchor I and Anchor II (shown in blue boxes) serve as the two key anchor points of the interface, while H-bonds in Labels 1–4 (shown in black boxes) are formed between residues from the α1-helix of the PD and the RBM motif and α3-helix of the RBD. The numbering these labels is based on the locations of these residues along α1-helix.

Using the trajectory of the last 150 ns and an occupancy threshold of 10%, the H-bond interactions at the binding interface of the RBD/PD complex were analyzed, and 19 pairs of H-bonds and 2 salt bridges were detected (Figure 2, Table S1). Among these, seven H-bonds and one salt bridge have been reported at the binding interface (Table S1, shown in bold and underlined) [8], and twelve H-bonds and one salt bridge were newly detected in the equilibrated complexes. Of all the 19 H-bonding events, 14 occurred on the α1-helix (S19-N51) from the PD, 1 occurred on the C-terminus of the α2-helix, and 4 occurred on the tip of the β3β4-hairpin (Figure 2). Two salt bridges were formed between D30 and K417, and between K31 and E484, with occupancies of 29.90% and 42.51%, respectively. After the initial structural adjustment, some previously observed H-bonds in the static complex decreased, whereas new ones were formed because of the dynamic changes at the binding interface [9]. Among the 19 H-bonds, 3 strong H-bonds were formed by T83-N487 (Figure 2, Anchor I), K353-G502 (Figure 2, Anchor II), and D355-T500, with their occupancies all above 60%. A stable binding interface was formed between the RBD and PD. The H-bonds involving the α1-helix were mainly weak and moderate (Figure 2, labeled 1–4), with occupancies varying from 10.36% to 47.01%, indicating that the α1-helix was more flexible or dynamic at the binding interface compared to the two stable anchor points. The collective findings indicate that the RBM motif of the RBD resembles a curved human palm cradling the α1-helix, α2-helix, and β3β4-hairpin of the PD. In addition to forming a stable binding interface, the intrinsically disordered nature of the RBM and the highly dynamic α1-helix may lay the foundation for high-pressure- and temperature-regulated changes in RBD/PD binding.

### 2.2. High Pressure Enhances RBD/PD Binding

**Figure 3:**
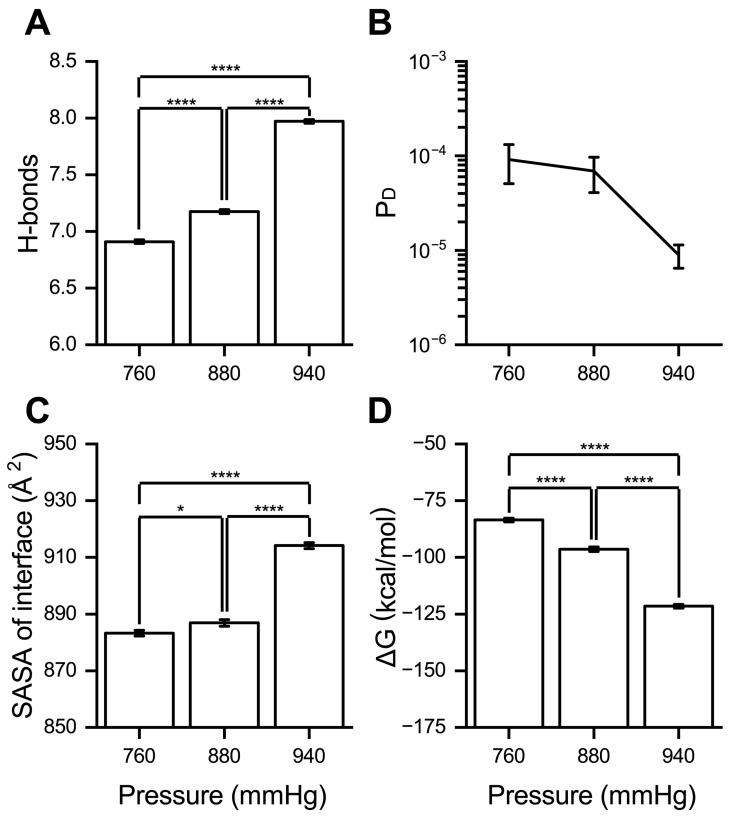
High pressure enhances RBD/PD binding. The average (**A**) number of H-bonds, (**B**) dissociation probability (P_D_), (**C**) SASA of the interface, and (**D**) binding energy (∆G, MM-PBSA) under different pressures (760, 880, and 940 mmHg) at a constant temperature of 310 K. Data are expressed as mean ± SEM. Statistical significance was analyzed using one-way ANOVA followed by Tukey’s multiple comparisons test. **** indicates *p* < 0.0001, and * indicates *p* < 0.05.

To investigate the impact of high pressure on RBD/PD binding, the stable complex conformations obtained at a baseline value of 760 mmHg (1 atm) were used. We incrementally increased the pressure to 1570 mmHg within ~15 ns and maintained it for 5 ns. Then, we released the pressure to 940 mmHg (760 + 180 mmHg, corresponding to Grade 3 hypertension pressure) and 880 mmHg (760 + 120 mmHg, corresponding to normal blood pressure) within ~2.0 ns and ~2.2 ns, respectively. Production runs were performed at 880 and 940 mmHg for 100 ns, with each condition replicated three times. As a control, production runs were conducted at the standard atmospheric pressure of 760 mmHg for 100 ns without pressure manipulation. The average number of H-bonds, dissociation probability (P_D_), SASA values, and binding energy (∆G) of the RBD/PD complex over 100 ns were analyzed as a function of system pressure. When the system pressure increased from the baseline value of 760 mmHg to the physiological blood pressure of 880 mmHg (760 + 120 mmHg), the number of H-bonds increased by approximately 0.26, the P_D_ decreased by approximately 24.42%, the SASA of the binding interface increased by approximately 3.63 Å^2^, and the ∆G decreased by approximately 12.90 kcal/mol (Figure 3A–D). These results suggest that the binding affinity of the RBD/PD complex was slightly enhanced under physiological blood pressure conditions compared to baseline pressure. When the system pressure was further increased to the Grade 3 hypertension level of 940 mmHg (760 + 180 mmHg), the number of H-bonds increased by approximately 1.06, the P_D_ decreased by approximately 9.22-fold, the SASA values of the binding interface increased by approximately 30.86 Å^2^, and the ∆G decreased by approximately 37.91 kcal/mol (Figure 3A–D). These findings indicated that the binding affinity between spike-RBD and ACE2-PD was significantly enhanced under high blood pressure conditions.

### 2.3. Curving of α1-Helix in the PD Mediates High-Pressure-Enhanced RBD/PD Binding

**Figure 4:**
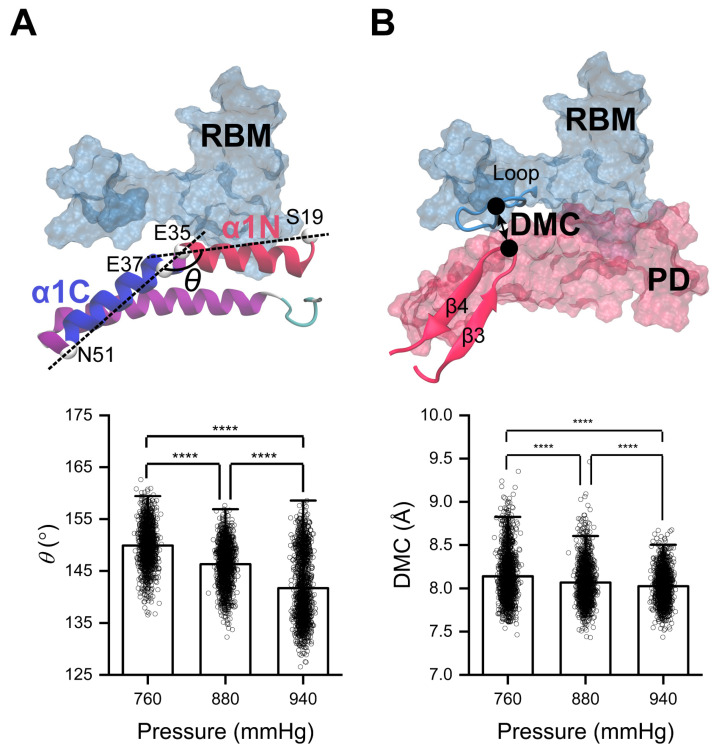
High pressure induces curving of the α1-helix and closing of the tip of the β3β4-hairpin to the RBM motif. (**A**) Angle θ between the N-terminus (α1N, residues S19-E35) and the C-terminus (α1C, residues E37-N51) of the α1-helix in the PD. (**B**) Distance of mass center (DMC) between the tip of β3β4-hairpin (red) of the PD and its interacting loop (cyan) from the RBM motif, a binding site of the RBD for the PD, under different pressures. Statistical significance was analyzed using one-way ANOVA followed by Tukey’s multiple comparisons test. **** indicates *p* < 0.0001. Effect size analyses were performed to compare the differences among the mean values, and Cohen’s |d| values ≥ 0.8 indicate a large effect size.

To elucidate the relationship between the structure and function of the high-pressure-induced enhancement in RBD/PD binding, we analyzed the trajectories of the RBD/PD complexes under normal physiological conditions (760 + 120 mmHg, 310 K) and high blood pressure conditions (760 + 180 mmHg, 310 K). Representative conformations were selected for further analysis. The binding interfaces of the RBD/PD complexes were compared by structural alignment. The major structural change occurred in the α1-helix on the outer surface of the PD. The α1-helix became more curved under high blood pressure, as reflected by the angle θ between its N-terminus (α1N, residues S19-E35) and C-terminus (α1C, residues E37-N51). The angle θ decreased from 146.30 ± 3.96° under normal blood pressure (760 + 120 mmHg) to 141.73 ± 6.46° under high blood pressure (760 + 180 mmHg) (Figure 4A). A minor structural change was also observed in the tip of the β3β4-hairpin of the PD, which became closer to its interacting loop of the RBM motif under high blood pressure. This is reflected by the slightly decreased distance of the mass center (DMC) between them, reducing from 8.07 ± 0.23 Å under normal blood pressure to 8.03 ± 0.18 Å under high blood pressure (Figure 4B).

**Figure 5:**
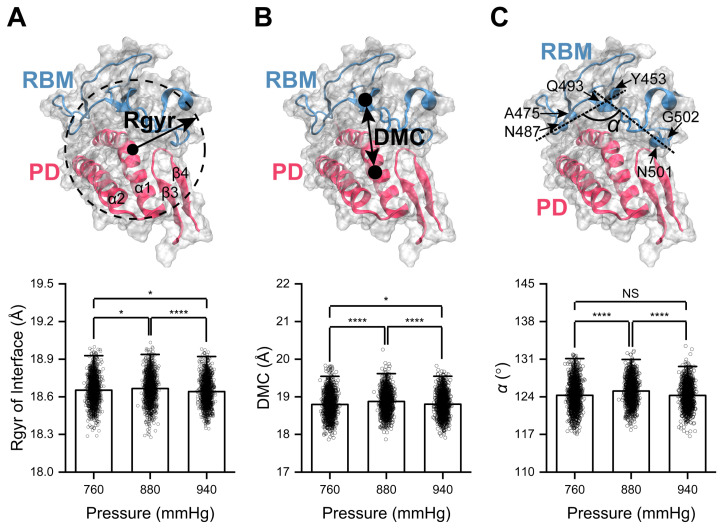
High pressure induces contraction of the binding interface of the RBD/PD complex. (**A**) Rgyr of the binding interface, including the RBM motif (cyan) of the spike-RBD domain and the α1-helix, α2-helix, β3β4-hairpin of the ACE2-PD domain (red). (**B**) Distance of mass center (DMC) between the RBM motif and the interaction surface of the PD. (**C**) Cross angle α between the two straight lines connecting the DMC of two sides (residues A475 and N487 on one side; residues N501 and G502 on the other side) to the middle region (residues Y453 and Q493) of the outer surface of the RBM motif, respectively, under different pressures and at a constant temperature of 310 K. Statistical significance was analyzed using one-way ANOVA followed by Tukey’s multiple comparisons test. **** indicates *p* < 0.0001, * indicates *p* < 0.05, and NS indicates not significant. Effect size analyses were performed to compare the differences among the mean values, and Cohen’s |d| values ≥ 0.8 indicate a large effect size.

The radius of gyration (Rgyr) is an indicator of the compactness of the protein structure and is used to describe changes in compactness at the binding interface of RBD/PD complexes under high pressure. Here, the secondary structures involved in binding interface forming were considered, including the RBM motif from the RBD and α1-helix/α2-helix/β3β4-hairpin from the PD. The average Rgyr of the binding interface under high pressure decreased from 18.67 ± 0.11 to 18.64 ± 0.10 Å, indicating a slight contracting of the binding interface under high pressure (Figure 5A). The DMC between the RBM motif and the surface of the PD (including α1-helix/α2-helix/β3β4-hairpin) was calculated. The average DMC decreased from 18.88 ± 0.29 Å under normal blood pressure to 18.80 ± 0.29 Å under high blood pressure, indicating a more compact binding interface under high pressure (Figure 5B, Table S2). Considering that the RBM motif looked like an irregularly curved human palm and that the curving of the palm might mediate the contracting of the binding interface, the cross angle α between the two straight lines connecting the DMC of two sides was calculated. The outer surface of the RBM under high pressure was slightly curved compared to that under normal blood pressure, as the average angle α of 124.26 ± 2.14° was smaller than that (125.08 ± 2.24°) under normal blood pressure (Figure 5C). The collective findings indicate that the curving of the α1-helix, closing of the tip of β3β4-hairpin to the RBM motif, and curving of the outer surface of the RBM motif under high pressure synergistically result in the slight compacting of the binding interface and, thus, an enhancement in RBD/PD binding.

### 2.4. Key Residues Underlying High-Pressure-Upregulated RBD/PD Binding

**Figure 6:**
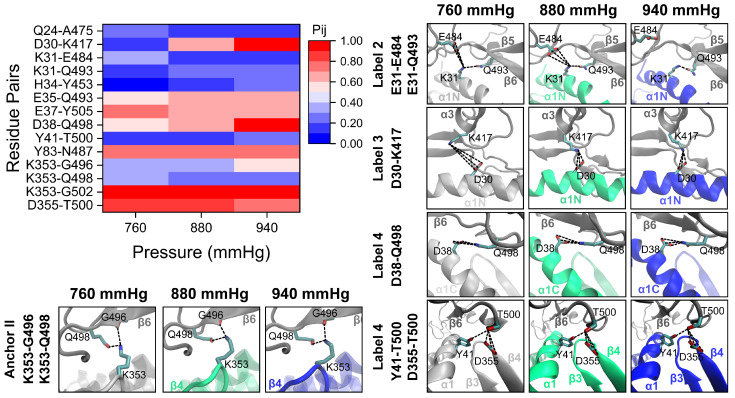
High pressure induces H-bond changes on the binding interface of RBD/PD complexes. The upper left displays the heatmap of the pairwise interaction probability (Pij) of residue pairs under different pressures and a constant temperature of 310 K. The Pij of the interacting residue pairs on the binding interface of RBD/PD complexes was calculated based on the occupancies of H-bonds (see Experimental Procedures). The boxes on the right and the bottom are enlarged views of the key H-bond events on the binding interface. Secondary structures from RBDs are drawn as gray NewCartoon, and secondary structures from the PDs under 760, 880, and 940 mmHg are drawn as silver, green, and blue NewCartoon, respectively. The residues involved are drawn in licorice. The enlarged views of labels 2–4 and Anchor II correspond to the labels in Figure 2. The residue pairs are marked on the left of the boxes. The H-bonds are shown as black dotted lines.

To further reveal the structural basis underlying the high-pressure-enhanced RBD/PD binding, the H-bond interactions of residue pairs forming the binding interface under normal blood pressure (880 mmHg) and high blood pressure (940 mmHg) were examined. The probabilities (Pij) of the residue pairs formed at the binding interface of the RBD/PD complexes were calculated based on the occupancies of the detected H-bonds, as described in our previous studies [43,44]. With a threshold of 10% for the Pij, 16 residue pairs were detected to form H-bonds at the binding interface of RBD/PD complexes with multifarious patterns in response to high pressure (Table S3). A heatmap of the Pij for the 14 key residue pairs is shown in Figure 6. For the anchor point involving the C-terminus of α2-helix (Figure 2, Anchor I), the Pij of Y83-N487 remained comparable under hypertensive and normotensive conditions, both exceeding 70%. The finding suggests that the stability of the anchor point was maintained at high blood pressure. For the other anchor point involving the tip of the β3β4-hairpin (Figure 2, Anchor II), the Pij of D355-T500 decreased slightly from over 80% to approximately 75%. However, the Pij of K353-Q498 and K353-G502 remained relatively stable at approximately 25% and 95%, respectively. The Pij of K353-G496 increased from <40% to >55%. Consequently, the hydrogen bonding interactions at this anchor point remained robust and were further strengthened under high pressure. This observation is consistent with the noted closure of the β3β4-hairpin tip towards the interacting loop from the RBM motif (Figure 4B). For residues involving the α1-helix (Figure 2, labeled 1–4) that underwent curving at A36 under high pressure (Figure 4A). The Pij of Q24-A475 and Q24-N487 (Figure 2, labeled 1), which is located at the N-terminus of α1N, only slightly changed and remained at low levels below 20%. The Pij of K31-E484, K31-Q493, and H34-Y453 (Figure 2, labeled 2), which is located at the C-terminus of α1N and forms weak H-bonds with the middle region of the outer surface of the RBM motif, did not show significant changes. The Pij of D30-K417 (Figure 2, labeled 3), which is located at the C-terminus of α1N, increased from >65% to >95%. The Pij of D38-Q498 (Figure 2, labeled 4), which is located at the N-terminus of α1C, increased from >60% to >95%. Altogether, when the blood pressure increased from a normal level of 880 mmHg to a high level of 940 mmHg, the two anchor points of the binding interface of the RBD/PD complex were further stabilized. High-pressure-induced curving of α1-helix led to a slight adjustment of the weak H-bonds and further enhancement in the strong H-bonds. This may provide a structural explanation for high-pressure-enhanced RBD/PD binding at the atomic level.

### 2.5. High Temperature Weakens and Low Temperature Enhances RBD/PD Binding

**Figure 7:**
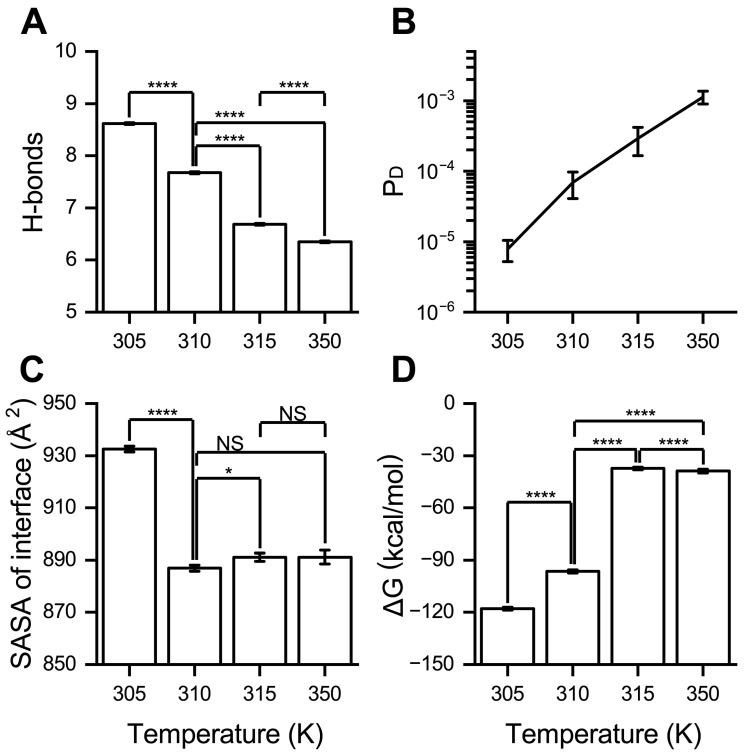
High temperature weakens and low temperature enhances RBD/PD binding. The average (**A**) number of H-bonds, (**B**) dissociation probability (P_D_), (**C**) SASA of the interface, and (**D**) binding energy (∆G, MM-PBSA) under different temperatures and a constant pressure of 880 mmHg (normal blood pressure). Data are shown as mean ± SEM. Statistical significance was analyzed using one-way ANOVA followed by Tukey’s multiple comparisons test. **** indicates *p* < 0.0001, * indicates *p* < 0.05, or NS indicates not significant.

To explore the effect of high fever temperatures on RBD/PD binding, the complex systems were annealed at four temperatures, i.e., 350 K (extremely high temperature), 315 K (the upper limit of fever temperature), 310 K (normal body temperature), and 305 K (close to the temperature of the nasal mucosa, a common entry point for viruses), to perform production runs for 100 ns with three replicates. Compared to the parameters under a normal body temperature of 310 K, when the temperature increased to the high fever level of 315 K, the number of H-bonds formed at the interfaces decreased by approximately 0.99, the P_D_ increased by approximately 3.23-fold, the binding energy ∆G decreased by approximately 61.28% (by about 59.12 kcal/mol), and the SASA of the binding interface increased by approximately 4.17 Å^2^ (Figure 7). These findings suggest that the binding affinity between the RBD and PD is significantly weakened at elevated temperatures associated with high fever. When the temperature was further increased to the extremely high level of 350 K, the number of hydrogen bonds decreased by approximately 1.32, ∆G reduced by approximately 59.70% (by about 57.59 kcal/mol), and P_D_ increased by approximately 15.38-fold. However, the SASA of the binding interface remained comparable to those observed at 310 and 315 K (Figure 7). These findings indicate that the temperature elevation to above 315 K significantly weakened RBD/PD binding. Conversely, when the temperature decreased from 310 K to 305 K, the number of H-bonds increased by approximately 0.94, P_D_ diminished by approximately 7.80-fold, ∆G rose by approximately 22.22% (by about 21.44 kcal/mol), and the SASA of the binding interface increased by approximately 45.62 Å^2^. These changes suggest that a lower temperature of 305 K enhances the binding affinity between the RBD and PD.

### 2.6. Straightening of the α1-Helix in the PD Mediates High-Temperature Weakening of RBD/PD Binding

**Figure 8:**
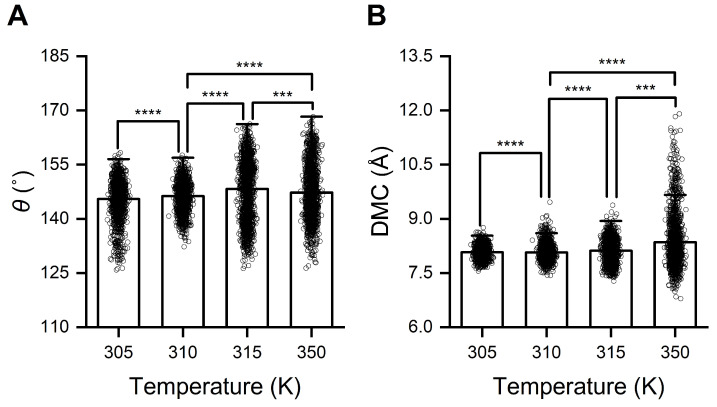
High temperature induces straightening of α1-helix and movement away of the tip of β3β4-hairpin from the RBM motif. (**A**) Angle θ between α1N and α1C. (**B**) The DMC between the tip of the β3β4-hairpin of the PD and its interacting loop from the RBM motif under different temperatures and a constant pressure of 880 mmHg. The calculation method is consistent with that provided in Figure 4. Statistical significance was analyzed using one-way ANOVA followed by Tukey’s multiple comparisons test. **** indicates *p* < 0.0001, and *** indicates *p* < 0.001. Effect size analyses were performed to compare the differences among the mean values, and Cohen’s |d| values ≥ 0.8 indicate a large effect size.

To elucidate the structural changes underlying temperature-regulated RBD/PD binding, we analyzed the trajectories of RBD/PD complexes at various temperatures (305, 315, and 350 K) under the same pressure (880 mmHg). Representative conformations were selected and compared with those observed at normal body temperature (310 K). Structural alignment revealed that the α1-helix in the PD became significantly straighter at high temperatures (315 and 350 K) compared to normal body temperature (310 K). Additionally, the tip of the β3β4-hairpin was observed to be positioned further away from the RBM under these high-temperature conditions. Calculating the angle θ between α1N and α1C of the α1-helix and the DMC between the tip of the β3β4-hairpin and its interacting loop from the RBM verified these structural changes. When the temperature was elevated to 315 K, the angle θ increased from 146.30 ± 3.96° at normal body temperature to 148.27 ± 8.14° (Figure 8A), while the DMC increased from 8.07 ± 0.23 Å to 8.12 ± 0.31 Å (Figure 8B). When the temperature further increased to 350 K, the angle θ and DMC increased to 147.27 ± 7.50° and 8.35 ± 0.73 Å, respectively. When the temperature dropped to 305 K, the average angle θ decreased to 145.49 ± 5.04°, and the DMC slightly increased to 8.08 ± 0.17 Å. Under high temperatures, both the angle θ and DMC fluctuated in wider ranges. Especially at the extremely high temperature of 350 K, the angle θ fluctuated from 126 to 168°, and the DMC fluctuated from 6.5 to 11.9 Å. Conversely, at a lower temperature of 305 K, both the angle θ and DMC fluctuated in narrower ranges, with the angle θ fluctuating from 126 to 158° and the DMC fluctuating from 7.6 to 8.8 Å. The collective findings indicate that, on the one hand, the α1-helix in the PD became highly flexible (or elastic) and straighter at high temperatures, which was unfavorable for forming stable interfaces with the irregular outer surface of the RBD. On the other hand, dramatic thermal movements induced by high temperatures caused the tip of the β3β4-hairpin to move away from its interacting loop in the RBM, directly destabilizing one of the anchor points of the RBD/PD interface. Conversely, low temperatures constrain these thermal movements and stabilize the interface.

**Figure 9:**
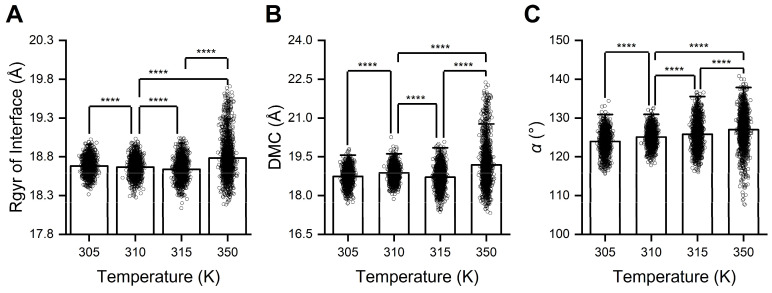
High temperature induces expansion, while low temperature induces compaction, of the binding interface of RBD/PD complexes. (**A**) Rgyr of the binding interface (here, considering the secondary structures involved in binding interface forming, that is, the RBM motif and α1-helix/α2-helix/β3-β4-hairpin from the PD). (**B**) DMC between the RBM motif and secondary structures from the PD. (**C**) Cross angle α between the two straight lines connecting the DMC of two sides to the middle region of the outer surface of the RBM motif, respectively, under different temperatures and a constant pressure of 880 mmHg. The calculation was performed as in Figure 5. Statistical significance was analyzed using one-way ANOVA followed by Tukey’s multiple comparisons test. **** indicates *p* < 0.0001. Effect size analyses were performed to compare the differences among the mean values, and Cohen’s |d| values ≥ 0.8 indicate a large effect size.

Rgyr was used to describe changes in the compactness of the binding interface of the RBD/PD complexes at high temperatures. The average Rgyr of the binding interface increased from 18.67 ± 0.11 Å at normal body temperature to 18.78 ± 0.25 Å at high temperatures and fluctuated within a wider range of 18.18–19.71 Å, indicating an expansion or loosening of the binding interface at high temperatures (Figure 9A). Calculating the DMC between the RBM motif and the interaction surface of the PD revealed an increase in the average DMC from 18.88 ± 0.29 Å at normal body temperature to 19.19 ± 0.82 Å at high temperature. The DMC at high temperatures fluctuated within a wider range of 17.32–22.38 Å, indicating a more separated and dynamic binding interface at high temperatures (Figure 9B, Table S4). The outer surface of the RBM motif became less curved at high temperatures, as reflected by the increased angle α of 127.01 ± 4.53° from 125.08 ± 2.24° at normal body temperature, and the angle α at high temperatures fluctuated within a wider range of 107.64–140.85° (Figure 9C). The collective findings indicate that at high temperatures, the straightening of the α1-helix, movement away of the tip of the β3β4-hairpin and the straightening of the outer surface of the RBM motif synergistically mediate the expansion or loosening of the binding interface of RBD/PD complexes.

### 2.7. Key Residues Underlying High-Temperature-Downregulated RBD/PD Binding

**Figure 10:**
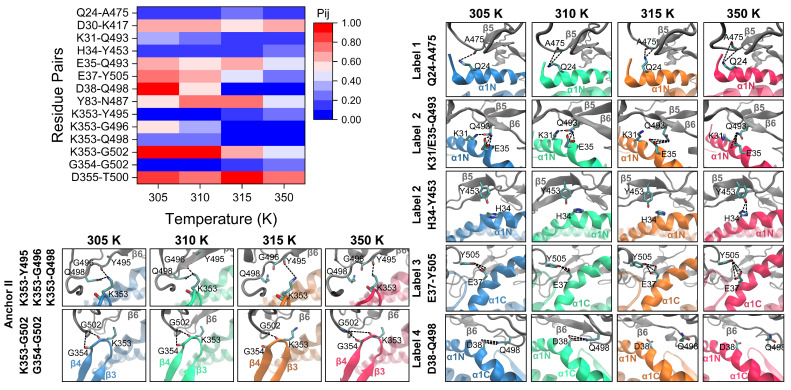
High temperature induces H-bonding changes on the binding interface of RBD/PD complexes. The upper left panel displays a heatmap of the Pij of the interacting residue pairs on the binding interface of RBD/PD complexes under different temperatures and a constant pressure of 880 mmHg, calculated based on the occupancies of H-bonds detected (see Experimental Procedures). The boxes on the right and the bottom are enlarged views of the key H-bonding events on the binding interface of RBD/PD complexes under different temperatures. Secondary structures from RBDs are drawn as gray NewCartoon. Secondary structures from PDs under 305 K, 310 K, 315 K, and 350 K are drawn as cyan-blue, green, orange, and red NewCartoon, respectively. The enlarged views of labels 1–4 and Anchor II correspond to the labels in Figure 2. The residues involved are drawn as licorice. The residue pairs are marked on the left of the boxes. The H-bonds are shown as black dotted lines.

To further elucidate the structural basis underlying temperature-regulated RBD/PD binding, we analyzed the H-bond interactions of the RBD/PD complexes at various temperatures (305, 315, and 350 K). We calculated and compared the Pij values of the residue pairs with those observed at normal body temperature. With a threshold of 10% for the Pij, a total of 20 residue pairs were identified (Table S5). Among these, six pairs exhibited weak instantaneous hydrogen bond interactions, with their Pij values remaining below 20% across all temperatures, which were disregarded. The remaining 14 residue pairs demonstrated a diverse temperature-dependent behavior in response to varying temperatures (Figure 10).

For the anchor point involving the C-terminus of the α2-helix (Figure 2 and Figure 10, Anchor I), the Pij values of Y83-N487 remained consistently above 60% at temperatures below 315 K. However, these values decreased to below 50% at 350 K, indicating that this anchor point is robust under minor temperature fluctuations but is significantly disrupted only at extremely high temperatures. For the other anchor point involving the tip of the β3β4-hairpin (Figure 2 and Figure 10, Anchor II), the Pij values for K353-G496 and K353-Q498 decreased to approximately 0% above 315 K, while that for K353-G502 gradually decreased from >90% to <50% at 350 K. The Pij values for K353-Y495 and G354-G502 gradually increased from approximately 0% to > 20% and 25%, respectively, at 350 K. The Pij values of these residue pairs increased when the temperature decreased to 305 K. The Pij value for D355-T500 remained at a high level of approximately 77% across all temperatures. These findings indicate that the anchor point undergoes substantial structural reorganization at high temperatures, resulting in partial destabilization. Conversely, low temperatures stabilize the anchor point. These observations align with the noted behavior of the β3β4-hairpin tip, which moved away from the RBM motif under high temperatures but approached it under low temperatures (Figure 8B). For residues involving the α1-helix (Figure 2 and Figure 10, label 1–4), straightening at high temperatures and curving at low temperatures (Figure 8A) were observed. The Pij of E35-Q493 (Figure 2 and Figure 10, label 2) decreased from approximately 60% to 40% at 350 K. The Pij of E37-Y505 (Figure 2 and Figure 10, label 3) gradually decreased with temperature elevation. The Pij of D30-K417 (Figure 2 and Figure 10, label 4) only slightly decreased at high temperatures. The Pij of D38-Q498 (Figure 2 and Figure 10, label 4) decreased from approximately 60% to almost 0% above 315 K. Conversely, when the temperature dropped to 305 K, all H-bonding interactions were enhanced to varying extents, as reflected by the increased Pij values for the involved residue pairs.

In summary, as the temperature increased from the normal body temperature of 310 K to above 315 K, the two anchor points, particularly the one involving the tip of the β3β4-hairpin, began to destabilize, leading to partial destabilization of the RBD/PD complex. Concurrently, the high-temperature-induced straightening of the α1-helix was detrimental to the cradling of the irregular concave outer surface of the RBM motif (Figure 8B), resulting in a significant weakening of strong hydrogen bonding interactions and minor adjustments in weak hydrogen bonding interactions. Conversely, at a lower temperature of 305 K, the two anchor points were stabilized, and the α1-helix exhibited increased curvature, forming a more compact binding interface (Figure 8A). These observations provide an atomic-level structural explanation for temperature-regulated binding of the RBD/PD complex.

## 3. Discussion

The COVID-19 global pandemic caused by SARS-CoV-2 has posed an unprecedented health crisis [1,2,3]. The efficient transmission and pathogenesis of SARS-CoV-2 rely on the initial recognition and binding of the RBD from the virus surface spike protein to the PD of the host cellular ACE2 receptor and subsequent conformational changes in the spike to form the fusion machinery [2,4,5]. Besides acting as a host cellular receptor for SARS-CoV-2, ACE2 is an endogenous counter-regulator of the RAAS and plays a critical role in blood pressure control [10,11], which provides a link between COVID-19 and RAAS.

Hypertension is a serious public health problem affecting approximately 31.1% of adults worldwide [15,16]. Hypertension is associated with a higher susceptibility to SARS-CoV-2 infection and increases the severity and fatality of COVID-19 [16,20,21,22,23,24,25,26]. The precise mechanism by which hypertension predisposes patients to unfavorable COVID-19 outcomes remains unclear. Since ACE2 is ubiquitously expressed in multiple organs, including the cardiovascular system [13,45,46], alterations in blood pressure might directly change the mechanical environment around ACE2 and thus affect its binding to the RBD. We hypothesized that the high blood pressure in hypertensive patients might enhance RBD/PD binding, leading to augmented attachment of the virus to human cells, accelerated viral progression, higher levels of viral load, and prolonged viral shedding.

Fever is a manifestation of a physical inflammatory response [34] and has been identified as a common symptom at the onset of SARS-CoV-2 infection [3,30,31,32,33]. A high temperature at the early stage of SARS-CoV-2 infection impairs binding to human cells and retards viral progression. A low body temperature at initial presentation is a marker of poor prognosis [35,38]. We hypothesized that high temperatures might weaken RBD/PD binding, leading to attenuated attachment of the virus to human cells, retarded viral progression, and lower levels of viral load, whereas low temperatures might otherwise enhance RBD/PD binding, as high pressure does.

To test these two hypotheses, all-atom MD simulations were performed under different pressures and temperatures to examine the effects of high pressure and temperature on RBD/PD binding. Our results suggest that a high blood pressure of 940 mmHg enhanced RBD/PD binding compared to a normal blood pressure of 880 mmHg, which was consistent with the hypothesis. The results also showed that temperatures above 315 K weakened RBD/PD binding, whereas a low temperature of 305 K enhanced RBD/PD binding. By disentangling the enthalpic (ΔH) and entropic contributions (TΔS) to the total binding energy (ΔG), it was found that RBD/PD binding was enthalpy-driven, and high pressure and low temperature favor the interaction (Table S10). These results were consistent with previous in vitro and in silico experimental findings [37,39,40,41,42]. For example, Finzi et al. utilized Isothermal Titration Calorimetry (ITC) and Biolayer Interferometry (BLI) to reveal that low temperatures of 22 °C (295 K) and 4 °C (277 K) significantly increased the binding of spike proteins (and RBDs) from variants of concern, including the Omicron variant, to ACE2 [40,41]. Our results also indicated that the curvature of the α1-helix from the PD and the proximity of the tip of the β3β4-hairpin of the PD to the RBM motif of the RBD governed the compactness of the binding interface and, thus, the binding affinity. Since the α1-helix is located on the outer surface of the lobe I subdomain of PD, anti-hypertensive drugs or small molecule inhibitors targeting the active site of hACE2, such as MLN-4760 [47], might lead to rearrangements of the α1-helix and its surrounding regions, thereby affecting RBD/PD binding. Investigating how common anti-hypertensive medications might affect the RBD/PD interaction through longer simulations could have clinical implications. In addition, there is currently a lack of direct experimental data supporting the pressure-regulated binding of spike (RBD)/PD, particularly within the range of hypertensive pressures. In future research, we could consider employing biophysical methods to quantify the impact of pressure on RBD/PD binding across a broader range of hypertensive conditions, validating our MD-predicted binding affinity changes and thereby filling this experimental data gap.

It is worth noting that hypertension is not merely a consequence of high blood pressure but also the combined effects of various factors, including cardiac output, vascular resistance, and vascular wall elasticity [27,28]. These factors collectively contribute to the complex pathophysiology of hypertension. In addition, we find that other biological factors, such as ACE2 expression and immune signaling, may also play a role in modulating this interaction [7,46]. Here, our study focused solely on isolating and examining the direct impact of elevated pressure on the binding characteristics of the RBD/PD complex, with specific attention to the mechanical and biophysical aspects, which provides a clearer understanding of the pressure-dependent effects on this interaction. In future studies, exploring the interplay between high blood pressure and other biological factors will provide a more comprehensive picture of how hypertension influences viral interactions at the molecular level. Similarly, while fever is a manifestation of complex physical inflammatory responses, our study focused on isolating and examining the direct impact of elevated temperature on RBD/PD binding. Meanwhile, it is crucial to emphasize that the extremely high temperature of 350 K serves solely as a control temperature for thermodynamic stability analysis. Its biological significance is confined to revealing temperature-sensitive critical phenomena, rather than simulating actual physiological environments.

The key residues governing high pressure and temperature-regulated RBD/PD binding were predicted and were further verified with the emergence of SARS-CoV-2 variants during the COVID-19 pandemic. For example, N487 formed a stable anchor point of the binding interface of the complex by interacting with Y83 from the PD, although direct N487 mutations were not observed in the emerging SARS-CoV-2 variants. Substitution of the nearby F486 to S486, P486, or V486 was observed in the Omicron-BA.2.75, Omicron-XBB, Omicron-BA.2.86, and Omicron-BA.5 lineages, respectively, and was confirmed to influence the binding affinity of RBD/PD [48]. The F486 mutation might alter the local environment of N487, thus changing the stability of this anchor point and the binding strength of RBD/PD. Furthermore, the tip of the β3β4-hairpin (covering residues L351-D355) from the PD constructed another anchor point of the binding interface via interactions with the β6α5-loop and α5-helix (covering residues Y495-Q506) from the RBD. Currently, T500, G502, G496, Q498, and Y495 are the key residues governing high-pressure- and temperature-regulated RBD/PD binding. N501 was located on the β6α5-loop of the RBD, and the N501Y substitution was a key mutation shared in the Alpha, Beta, and Gamma SARS-CoV-2 variants, as well as all the Omicron SARS-CoV-2 variants [48,49,50,51,52], and considerably enhanced RBD/PD binding. Since N501 was close to these predicted key residues, it was reasonable to speculate that the N501Y substitution might directly influence the stability of this anchor point and, thus, the binding interface. Meanwhile, Q498R, Y505H, and N501Y were three conserved mutations observed in all Omicron variants that could influence the binding affinity of RBD/PD [48,52,53,54,55,56], further verifying the importance of these residues in governing RBD/PD binding. Q493 was a key residue predicted to govern RBD/PD binding, and Q493R and Q493E were observed in Omicron-BA.2 variants and Omicron-KP.3 variants, respectively, which directly influenced binding affinity [57]. As an initial exploratory study, our work focused exclusively on the interaction between the WT SARS-CoV-2 RBD and hACE2-PD, elucidating how high blood pressure and temperature influence their binding. Throughout the COVID-19 pandemic, SARS-CoV-2 has continuously evolved, giving rise to numerous variants with distinct characteristics. In the near future, systematically investigating how pressure and temperature modulate the interactions between the RBDs of different SARS-CoV-2 variants and hACE2 across wider ranges of physiologically plausible hypertension pressures and temperatures will hold significant physiological relevance and transformative clinical implications. This is particularly crucial for determining whether the pressure- and temperature-dependent RBD/PD bindings exhibit variant-specific characteristics. Such insights would not only provide valuable guidance for treating hypertension patients infected with different variants but also deepen our understanding of the complex mechanisms underlying SARS-CoV-2 infection. Moreover, the spike protein is the outermost structural protein of the SARS-CoV-2 virus, which interacts with the human ACE2 receptor and facilitates entry into the respiratory system. Existing evidence indicates that only the RBDs in the spike protein can be recognized and bound by hACE2 [4,58,59,60,61]. The dynamic conformational transitions of the spike protein could affect the binding of spike to hACE2, and thus the entry of the SARS-CoV-2 virus. Simulating the binding of full-length spike and full-length hACE2 (in a lipid bilayer model) would provide a more comprehensive elucidation of the spike and hACE2 interactions within a cellular context, thus deepening our understanding of SARS-CoV-2 infection.

In conclusion, high blood pressure and low temperature enhanced RBD/PD binding, while high temperature significantly weakened this binding. The findings are expected to provide novel insights into the underlying mechanisms by which hypertension predisposes patients to unfavorable outcomes in COVID-19 and how an initial high temperature retards viral progression. The identified key residues governing high-pressure- and temperature-regulated RBD/PD binding could be used to design targeted drugs for hypertensive patients.

## 4. Materials and Methods

### 4.1. System Setup

The crystal structure of the RBD/PD complex, the minimal binding unit between the SARS-CoV-2 spike protein and human ACE2 receptor, was retrieved from the RCSB Protein Data Bank (PDB ID: 6M0J) [8]. The complex system, comprising the RBD (chain E, residues T333-G526), the PD (chain A, residues S19-D615), a zinc ion (Zn^2+^) bound to the PD, and five N-linked glycans (N53, N90, N322, and N546 and one with the RBD, N343), was built using the CHARMM-GUI Webserver [62,63]. The complex system was solvated with TIP3P water molecules in a rectangular box, ensuring that the box walls were at least 12 Å away from any protein atom. Sodium and chloride ions were added into the water box to achieve charge neutrality and simulate a physiological environment, resulting in a system containing approximately 106,000 atoms.

### 4.2. Minimization and Equilibrium of Complex Systems

The Visual Molecular Dynamics software package (VMD, Version 1.9.3) was used for visualization, modeling, and data analysis [64]. The Nanoscale Molecular Dynamics software package (NAMD, Version 2.13) [65,66] was used for MD simulations. The CHARMM36 all-atom force field [67], along with a cAMP correction for the backbone, the particle mesh Ewald algorithm for electrostatic interaction, and a 12 Å cutoff for electrostatic and van der Waals interaction, was used to perform MD simulations with periodical boundary conditions and a time step of 2 fs. Atomic coordinates were recorded every picosecond. The system was subjected to energy minimization following four steps. First, proteins, glycans, and Zn^2+^ were fixed, while water and neutralizing ions could move for 15,000 steps. Second, the backbone of proteins, glycans, and Zn^2+^ were fixed, while the sidechains, water molecules, and neutralizing ions were free to move for 15,000 steps. Third, the proteins were fixed, except for the glycan linking and Zn^2+^ ion coordinating residues, while the glycans and their linking residues, Zn^2+^ and its coordinating residues, water, and neutralizing ions were free to move for 15,000 steps. Fourth, the whole system was free to move for 15,000 steps. The energy-minimized system was heated gradually from 0 to 310 K within 100 ps and equilibrated first under an NVT ensemble (310 K) for 0.5 ns and then transformed to the NPT ensemble for 200 ns to obtain stable conformations of the RBD/PD complex. The temperature was held at 310 K using Langevin dynamics, and the pressure was maintained at 1 atm using the Langevin piston method.

### 4.3. Pressure and Temperature Simulations

To characterize the binding of RBD/PD complexes under normal and hypertensive blood pressure conditions, we simulated stable complex conformations obtained at 1 atm (760 mmHg) and 310 K. The system pressure was incrementally increased from the baseline value of 760 to 1570 mmHg within approximately 15 ns and was maintained at this elevated pressure for an additional 5 ns. Subsequently, the pressure was rapidly reduced to 940 mmHg (corresponding to Grade 3 hypertension [68]) and 880 mmHg (representative of normal blood pressure) within approximately 2.0 and 2.2 ns, respectively. Production runs were performed for 100 ns at 940 and 880 mmHg, each with three replicates. As a control, production runs were performed three times for 100 ns at a baseline value of 760 mmHg without pressure modulation of the system.

To describe the binding behavior of the RBD/PD complexes at different temperatures, we utilized the RBD/PD complex conformations obtained at 310 K and 880 mmHg as the starting conformations. The system was first heated to an extremely high temperature of 450 K within approximately 15.5 ns and maintained at this temperature for 5 ns, then annealed to a high temperature of 350 K (an extremely high temperature) and a high fever temperature of 315 K (upper limit of fever temperature) within ~1.9 and ~2.7 ns, respectively. The system was annealed directly from 310 K to a low temperature of 305 K (approximately the temperature of the nasal cavity, which is a common entry point of the virus) within ~0.1 ns to reveal the effect of low temperature on RBD/PD binding. The production runs were performed using an NPT ensemble (350 K/880 mmHg, 315 K/880 mmHg, and 305 K/880 mmHg) for 100 ns.

### 4.4. Data Analyses

Most data analyses used the VMD package and built-in plugins [64]. Structural clustering and inspection were performed using USCF Chimera [69] and VMD. The Cα-RMSD, nonbonded interaction energy (in vacuum, including electrostatic energy and van der Waals energy), and the buried Solvent Accessible Surface Area (here referred to as the SASA of the interface) were used to characterize the conformational changes and stability of the RBD/PD complex. A H-bond was characterized by a donor–acceptor distance threshold ≤3.5 Å and a donor–hydrogen–acceptor angle ≤30°. For the identification of a salt bridge, the criterion was a distance ≤3.5 Å between any oxygen atom of acidic residues (Asp or Glu) and nitrogen atoms of basic residues (Lys or Arg). To define a salt bridge, the criterion was a distance ≤3.5 Å between any oxygen atom of acidic residues (Asp or Glu) and nitrogen atoms of basic residues (Lys or Arg). The number of H-bonds formed at the binding interface was used to evaluate the binding strength of the RBD/PD complexes. The occupancy of a H-bond or salt bridge was defined as the percentage of simulation time during which these interactions were maintained. A H-bond with occupancy exceeding 50% (and for a salt bridge exceeding 10%) was considered stable. The occupancy of H-bonds was used to calculate the pairwise interaction Pij of the residue pairs involved in RBD/PD binding and the P_D_ of the complex, as described in our previous studies [43,44]. Briefly, the Pij predicts the significance of individual residue pairs involved in complex binding, whereas the P_D_ predicts the binding strength of complexes under varying pressures and temperatures. The interaction energies (∆G) for the RBD/PD complexes were calculated using the MM-PBSA implicit solvent method [70]. The MM energy was calculated using the NamdEnergy plugin integrated within the VMD [65,66], and the PB energy was calculated using Delphi [71]. The enthalpic and entropic contributions to RBD/PD binding were calculated using MolAICal [72] and Carma [73].

Since the α1-helix in the PD curved around residue A36, its bending under conditions of elevated pressure and temperature was quantified by calculating the cross angle θ between the N-terminus (α1N, residues S19-E35) and C-terminus (α1C, residues E37-N51) of the α1-helix. The DMC between the tip of the β3β4-hairpin and its interacting loop from the RBM motif was calculated to characterize their binding affinity.

### 4.5. Statistical Analysis

The data were analyzed using unpaired Student’s *t*-test or one-way analysis of variance with a multiple comparison test. Values were considered statistically significant at *p* < 0.05. Effect size analyses were also performed by calculating Cohen’s d values to elucidate the conformational changes with pressure and temperature variations. Cohen’s |d| values < 0.2 were considered to indicate a small effect size, 0.2 ≤ Cohen’s |d| values < 0.8 were considered to indicate a medium effect size, and Cohen’s |d| values > 0.8 were considered to indicate a large effect size.

## Data Availability

The crystal structure used in this study is available from the Protein Data Bank (PDB) with the following ID: 6M0J (https://www.rcsb.org/structure/6M0J) (accessed on 23 April 2023). The RBD/PD complex and the N-glycans were built with CHARMM-GUI Webserver (https://charmm-gui.org/, JOB ID: 8282619496, accessed on 30 April 2023). Molecular dynamics (MD) simulations were performed using NAMD (Version 2.13, https://www.ks.uiuc.edu/Research/namd/) (accessed on 15 December 2018). Molecular visualization/inspection and data analyses were performed using VMD (Version 1.9.3, https://www.ks.uiuc.edu/Research/vmd/) (accessed on 15 December 2018). Structure clustering and inspection were performed using USCF Chimera (Version 1.16, https://www.cgl.ucsf.edu/chimera/) (accessed on 17 March 2023). The CHARMM36 force field used in this study is available at https://www.charmm.org/ (accessed on 7 April 2019).

## Supplementary Materials

The following supporting information can be downloaded at https://www.mdpi.com/article/10.3390/ijms26073250/s1.
