# Supplementary material for: Molecular Basis of High-Blood-Pressure-Enhanced and High-Fever-Temperature-Weakened Receptor-Binding Domain/Peptidase Domain Binding: A Molecular Dynamics Simulation Study
Source: Int J Mol Sci. 2025 Mar 31;26(7):3250. doi: 10.3390/ijms26073250 (PMC11989460; doi:10.3390/ijms26073250)
Supplement: Supplementary file 1 [file ijms-26-03250-s001.zip › ijms-3532902-supplementary.pdf]

Supplementary Materials

Molecular Basis of High-Blood-Pressure-Enhanced and High-Fever-Temperature-Weakened Receptor-Binding Domain/Peptidase Domain Binding: A Molecular Dynamics Simulation Study

Xubin Xie [†], Yu Zhang [†], Ying Fang, Jianhua Wu * and Quhuan Li *

Table S1. Major H-bonds and salt bridges formed at the binding interface of the equilibrated RBD/PD complex.

Number	PD (ACE2)	RBD (SARS-CoV-2)	Occupancy (%)
1	(Q)Gln^{24}-NE2	(A)Ala^{475}-O	13.45
2	(Q)Gln^{24}-OE1	(N)Asn^{487}-ND2	11.25
3	(D)Asp^{30}-OD2	(K)Lys^{417}-NZ	42.29
4	(K)Lys^{31}-NZ	(E)Glu^{484}-OE2	11.47
5	(K)Lys^{31}-NZ	(E)Glu^{484}-OE1	10.36
6	(K)Lys^{31}-NZ	(Q)Gln^{493}-OE1	15.19
7	(E)Glu^{35}-OE1	(Q)Gln^{493}-NE2	27.29
8	(E)Glu^{35}-OE2	(Q)Gln^{493}-NE2	24.19
9	(E)Glu^{35}-CD	(Q)Gln^{493}-NE2	12.00
10	(E)Glu^{37}-OE2	(Y)Tyr^{505}-OH	34.25
11	(E)Glu^{37}-OE1	(Y)Tyr^{505}-OH	26.08
12	(D)Asp^{38}-OD2	(Q)Gln^{498}-NE2	47.01
13	(D)Asp^{38}-OD1	(Q)Gln^{498}-NE2	31.84
14	(Y)Tyr^{41}-OH	(T)Thr^{500}-OG1	13.23
15	(Y)Tyr^{83}-OH	(N)Asn^{487}-OD1	68.49
16	(K)Lys^{353}-NZ	(G)Gly^{496}-O	42.49
17	(K)Lys^{353}-NZ	(Q)Gln^{498}-OE1	19.73
18	(K)Lys^{353}-O	(G)Gly^{502}-N	83.57
19	(D)Asp^{355}-OD2	(T)Thr^{500}-OG1	66.88
#	(D)Asp^{30}	(K)Lys^{417}	29.90
#	(K)Lys^{31}	(E)Glu^{484}	42.51

Notes：# indicates salt bridges formed at the interface of the RBD/PD complex. The H-bond occupancy threshold is set to > 10%. The H-bonds and salt bridges that have been reported in previous studies are shown in bold and underlined. One-letter abbreviations for amino acids are provided in parentheses.

Table S2. The intermolecular donor-acceptor distances for H-bonds formed at the binding interface of RBD/PD complexes under different pressures and a constant temperature of 310 K.

Number	PD (ACE2)	RBD (SARS-CoV-2)	Distance (Å)		
			760 mmHg	880 mmHg	940 mmHg
1	(Q)Gln^{24}-NE2	(A)Ala^{475}-C	5.2±0.9	4.8±1.0	4.6±1.1
2	(Q)Gln^{24}-NE2	(A)Ala^{475}-O	5.2±0.9	4.8±1.0	4.6±1.1
3	(Q)Gln^{24}-OE1	(N)Asn^{487}-ND2	3.5±0.7	3.7±0.9	3.6±0.8
4	(D)Asp^{30}-CG	(K)Lys^{417}-NZ	7.7±1.8	5.1±2.3	3.7±0.6
5	(D)Asp^{30}-OD1	(K)Lys^{417}-NZ	7.8±2.0	5.4±2.1	4.2±0.7
6	(D)Asp^{30}-OD2	(K)Lys^{417}-NZ	7.7±1.8	4.6±2.7	2.9±0.8
7	(K)Lys^{31}-NZ	(E)Glu^{484}-CD	5.4±1.4	6.2±2.1	6.2±2.0
8	(K)Lys^{31}-NZ	(E)Glu^{484}-OE1	5.1±1.6	5.9±2.3	6.0±2.2
9	(K)Lys^{31}-NZ	(E)Glu^{484}-OE2	5.2±1.6	5.9±2.3	5.8±2.3
10	(K)Lys^{31}-NZ	(Q)Gln^{493}-OE1	4.6±1.7	4.1±1.5	4.5±1.7
11	(H)His^{34}-NE2	(Y)Lys^{417}-NZ	4.0±1.3	5.0±1.4	5.5±0.9
12	(H)His^{34}-NE2	(Y)Tyr^{453}-OH	4.3±0.5	4.4±0.6	4.6±0.6
13	(H)His^{34}-ND1	(Y)Tyr^{453}-OH	4.5±0.6	4.1±0.8	3.8±0.7
14	(E)Glu^{35}-CD	(Q)Gln^{493}-NE2	3.9±0.6	4.1±0.8	4.0±0.7
15	(E)Glu^{35}-OE1	(Q)Gln^{493}-NE2	4.1±1.0	4.3±1.2	4.2±1.0
16	(E)Glu^{35}-OE2	(Q)Gln^{493}-NE2	4.1±1.0	4.2±1.1	4.2±1.1
17	(E)Glu^{37}-CD	(Q)Tyr^{505}-OH	4.1±1.0	4.1±0.9	4.0±0.9
18	(E)Glu^{37}-OE1	(Y)Tyr^{505}-OH	3.8±1.1	3.6±1.0	3.6±1.1
19	(E)Glu^{37}-OE2	(Y)Tyr^{505}-OH	3.7±1.1	3.8±1.0	3.8±1.1
20	(D)Asp^{38}-CG	(G)Gln^{498}-NE2	5.0±2.1	5.1±2.1	3.9±0.8
21	(D)Asp^{38}-OD1	(Q)Gln^{498}-NE2	5.5±2.1	5.5±2.2	4.7±0.8
22	(D)Asp^{38}-OD2	(Q)Gln^{498}-NE2	4.5±2.3	4.9±2.4	3.3±1.2
23	(Y)Tyr^{41}-OH	(T)Thr^{500}-OG1	3.0±0.2	3.0±0.2	3.0±0.2
24	(Y)Tyr^{83}-OH	(NAsn^{487}-OD1	2.8±0.3	3.0±0.6	2.9±0.6
25	(Y)Tyr^{83}-OH	(N)Asn^{487}-ND2	4.5±0.4	4.5±0.4	4.5±0.4
26	(K)Lys^{353}-NZ	(G)Tyr^{495}-O	6.9±0.4	7.0±0.6	6.8±0.2
27	(K)Lys^{353}-NZ	(G)Gly^{496}-O	3.6±1.6	3.4±1.2	2.8±0.4
28	(K)Lys^{353}-NZ	(Q)Gln^{498}-OE1	3.7±2.0	4.6±2.9	2.9±0.4
29	(K)Lys^{353}-O	(G)Gly^{502}-N	3.0±0.2	3.0±0.2	2.9±0.2
30	(G)Gly^{354}-O	(G)Gly^{502}-N	3.6±0.4	3.6±0.3	3.6±0.3
31	(D)Asp^{355}-CG	(T)Thr^{500}-OG1	3.9±0.6	4.0±0.6	4.1±0.6
32	(D)Asp^{355}-OD1	(T)Thr^{500}-OG1	3.6±1.1	4.6±0.7	4.6±0.7
33	(D)Asp^{355}-OD2	(T)Thr^{500}-OG1	3.9±0.8	3.0±0.6	3.1±0.6

Note: One-letter abbreviations for amino acids were provided in parentheses.

Table S3. Probabilities (Pij) of the residue pairs forming H-bonds on the binding interface of RBD/PD complexes under different pressures and a constant temperature of 310 K (with Pij > 10%).

Number	PD (ACE2)	RBD (SARS-CoV-2)	Pij (%)		
			**760 mmHg**	**880 mmHg**	**940 mmHg**
1	(Q)Gln^{24}	(A)Ala^{475}	18.69±25.58	10.57±10.22	14.27±6.47
2	(Q)Gln^{24}	(N)Asn^{487}	13.92±6.34	10.93±2.96	7.66±1.32
3	(D)Asp^{30}	(K)Lys^{417}	10.72±18.57	56.59±31.49	79.30±4.51
4	(K)Lys^{31}	(E)Glu^{484}	29.96±21.52	11.93±12.89	13.21±12.07
5	(K)Lys^{31}	(Q)Gln^{493}	13.29±6.53	19.19±10.67	19.11±6.41
6	(H)His^{34}	(K)Lys^{417}	14.15±2.57	6.97±12.07	0.00±0.00
7	(H)His^{34}	(Y)Tyr^{453}	2.03±3.51	10.89±6.25	16.91±2.57
8	(E)Glu^{35}	(Q)Gln^{493}	48.72±3.76	51.67±1.12	51.73±6.58
9	(E)Glu^{37}	(Y)Tyr^{505}	57.94±2.33	56.59±7.24	57.26±4.51
10	(D)Asp^{38}	(Q)Gln^{498}	42.57±33.79	50.47±27.14	79.70±13.56
11	(Y)Tyr^{41}	(T)Thr^{500}	11.39±15.18	16.51±6.83	18.29±9.60
12	(Y)Tyr^{83}	(N)Asn^{487}	62.21±10.62	61.73±9.18	61.29±10.57
13	(K)Lys^{353}	(G)Gly^{496}	25.30±22.12	32.51±15.04	46.03±5.73
14	(K)Lys^{353}	(Q)Gln^{498}	25.27±6.44	19.75±7.17	23.10±0.92
15	(K)Lys^{353}	(G)Gly^{502}	82.73±2.07	79.77±1.68	82.17±0.15
16	(D)Asp^{355}	(T)Thr^{500}	72.95±14.39	66.93±6.65	62.26±11.51

Note: One-letter abbreviations for amino acids were provided in parentheses.

Table S4. The intermolecular donor-acceptor distances for H-bonds formed at the binding interface of RBD/PD complexes under different temperatures and a constant pressure of 880 mmHg.

Number	PD (ACE2)	RBD (SARS-CoV-2)	Distance (Å)			
			305 K	310 K	315 K	350 K
1	(Q)Gln^{24}-NE2	(A)Ala^{475}-C	4.8±1.4	4.8±1.0	4.3±1.2	5.1±1.6
2	(Q)Gln^{24}-NE2	(A)Ala^{475}-O	4.8±1.4	4.8±1.0	4.3±1.2	5.1±1.6
3	(Q)Gln^{24}-OE1	(N)Asn^{487}-ND2	4.4±1.2	3.7±0.9	4.2±1.4	5.4±1.9
4	(D)Asp^{30}-CG	(K)Lys^{417}-NZ	4.5±1.9	5.1±2.3	5.4±2.3	4.7±2.0
5	(D)Asp^{30}-OD1	(K)Lys^{417}-NZ	4.2±2.0	5.4±2.1	5.5±2.3	4.7±2.1
6	(D)Asp^{30}-OD2	(K)Lys^{417}-NZ	4.4±2.0	4.6±2.7	5.1±2.6	4.5±2.1
7	(K)Lys^{31}-NZ	(E)Glu^{484}-CD	7.8±3.3	6.2±2.1	11.7±3.4	11.5±4.0
8	(K)Lys^{31}-NZ	(E)Glu^{484}-OE1	7.6±3.6	5.9±2.3	11.9±3.7	11.7±4.3
9	(K)Lys^{31}-NZ	(E)Glu^{484}-OE2	7.7±3.5	5.9±2.3	12.0±3.7	11.8±4.3
10	(K)Lys^{31}-NZ	(Q)Gln^{493}-OE1	4.0±1.7	4.1±1.5	4.1±1.6	4.7±2.0
11	(H)His^{34}-NE2	(Y)Lys^{417}-NZ	5.1±1.1	5.0±1.4	5.4±1.4	6.4±1.9
12	(H)His^{34}-NE2	(Y)Tyr^{453}-OH	4.6±0.5	4.4±0.6	4.5±0.6	3.9±0.9
13	(H)His^{34}-ND1	(Y)Tyr^{453}-OH	3.9±0.7	4.1±0.8	4.2±0.9	4.2±0.8
14	(E)Glu^{35}-CD	(Q)Gln^{493}-NE2	3.9±0.6	4.1±0.8	4.0±0.7	4.7±1.3
15	(E)Glu^{35}-OE1	(Q)Gln^{493}-NE2	4.0±1.0	4.3±1.2	4.2±1.1	4.9±1.5
16	(E)Glu^{35}-OE2	(Q)Gln^{493}-NE2	4.0±1.0	4.2±1.1	4.1±1.1	4.9±1.5
17	(E)Glu^{37}-CD	(Q)Tyr^{505}-OH	3.8±0.5	4.1±0.9	5.2±1.8	5.8±1.8
18	(E)Glu^{37}-OE1	(Y)Tyr^{505}-OH	3.6±0.8	3.6±1.0	4.9±1.8	5.5±1.8
19	(E)Glu^{37}-OE2	(Y)Tyr^{505}-OH	3.4±0.8	3.8±1.0	4.9±1.8	5.5±1.8
20	(D)Asp^{38}-CG	(G)Gln^{498}-NE2	3.8±0.2	5.1±2.1	7.8±1.0	8.0±1.5
21	(D)Asp^{38}-OD1	(Q)Gln^{498}-NE2	3.1±0.5	5.5±2.2	7.8±1.2	7.9±1.7
22	(D)Asp^{38}-OD2	(Q)Gln^{498}-NE2	4.6±0.5	4.9±2.4	7.8±1.3	7.9±1.6
23	(Y)Tyr^{41}-OH	(T)Thr^{500}-OG1	3.0±0.2	3.0±0.2	3.3±0.5	4.0±1.6
24	(Y)Tyr^{83}-OH	(N)Asn^{487}-OD1	3.3±0.9	3.0±0.6	3.1±0.9	4.0±1.8
25	(Y)Tyr^{83}-OH	(N)Asn^{487}-ND2	4.3±0.7	4.5±0.4	4.5±0.7	4.8±1.6
26	(K)Lys^{353}-NZ	(G)Tyr^{495}-O	6.8±0.2	7.0±0.6	4.1±1.0	4.8±2.0
27	(K)Lys^{353}-NZ	(G)Gly^{496}-O	2.8±0.4	3.4±1.2	4.9±1.1	4.9±1.3
28	(K)Lys^{353}-NZ	(Q)Gln^{498}-OE1	2.8±0.1	4.6±2.9	8.0±1.1	7.8±1.8
29	(K)Lys^{353}-O	(G)Gly^{502}-N	2.9±0.2	3.0±0.2	3.5±1.2	4.1±1.3
30	(G)Gly^{354}-O	(G)Gly^{502}-N	3.6±0.3	3.6±0.3	3.6±0.5	3.7±1.1
31	(D)Asp^{355}-CG	(T)Thr^{500}-OG1	3.8±0.5	4.0±0.6	3.7±0.5	4.4±1.7
32	(D)Asp^{355}-OD1	(T)Thr^{500}-OG1	4.4±0.5	4.6±0.7	4.3±0.5	4.6±1.5
33	(D)Asp^{355}-OD2	(T)Thr^{500}-OG1	2.9±0.4	3.0±0.6	2.8±0.5	4.0±2.2

Note: One-letter abbreviations for amino acids were provided in parentheses.

Table S5. Probabilities (Pij) of the residue pairs forming H-bonds on the binding interface of RBD/PD complexes at different temperatures and a constant pressure of 880 mmHg (with Pij > 10%).

**Number**	**PD (ACE2)**	**RBD (SARS-CoV-2)**	**Pij (%)**			
			305 K	310 K	315 K	350 K
1	(S)Ser^{19}	(A)Ala^{475}	10.16±4.48	3.65±6.33	0.00±0.00	0.00±0.00
2	(Q)Gln^{24}	(Q)Gln^{474}	10.41±10.94	0.00±0.00	0.00±0.00	0.00±0.00
3	(Q)Gln^{24}	(A)Ala^{475}	16.97±15.56	10.57±10.22	24.16±7.81	12.75±2.94
4	(Q)Gln^{24}	(N)Asn^{487}	6.57±11.38	10.93±2.96	9.12±9.93	5.49±5.45
5	(D)Asp^{30}	(K)Lys^{417}	60.39±19.87	56.59±31.49	49.30±32.76	52.53±10.84
6	(K)Lys^{31}	(E)Glu^{484}	8.24±9.29	11.93±12.89	0.00±0.00	7.00±12.13
7	(K)Lys^{31}	(Q)Gln^{493}	28.30±3.96	19.19±10.67	16.11±3.09	16.63±6.71
8	(H)His^{34}	(Y)Tyr^{453}	15.59±10.65	10.89±6.25	9.75±8.68	17.43±4.75
9	(E)Glu^{35}	(Q)Gln^{493}	56.62±3.30	51.67±1.12	56.08±5.30	36.86±3.47
10	(E)Glu^{37}	(Y)Tyr^{505}	64.39±2.96	56.59±7.24	35.07±16.85	19.24±12.02
11	(D)Asp^{38}	(Q)Gln^{498}	86.56±4.26	50.47±27.14	0.00±0.00	0.00±0.00
12	(Y)Tyr^{41}	(T)Thr^{500}	6.12±5.55	16.51±6.83	1.83±3.16	6.45±0.64
13	(Y)Tyr^{83}	(N)Asn^{487}	50.28±17.58	61.73±9.18	64.09±8.23	41.02±8.04
14	(Y)Tyr^{83}	(Y)Tyr^{489}	0.00±0.00	0.00±0.00	12.67±21.95	9.65±6.00
15	(K)Lys^{353}	(Y)Tyr^{495}	0.00±0.00	0.00±0.00	14.47±25.06	19.97±15.76
16	(K)Lys^{353}	(G)Gly^{496}	44.80±1.47	32.51±15.04	0.00±0.00	0.00±0.00
17	(K)Lys^{353}	(Q)Gln^{498}	24.81±0.96	19.75±7.17	0.00±0.00	0.00±0.00
18	(K)Lys^{353}	(G)Gly^{502}	84.81±0.50	79.77±1.68	60.14±3.32	41.14±17.44
19	(G)Gly^{354}	(G)Gly^{502}	0.00±0.00	0.00±0.00	15.20±26.33	24.52±19.24
20	(D)Asp^{355}	(T)Thr^{500}	75.91±3.40	66.93±6.65	84.56±0.66	67.22±16.48

Note: One-letter abbreviations for amino acids were provided in parentheses.

Table S6. Effect size analyses for the Angle θ and DMC presented in Figure 4.

**Cohen's d**		Angle θ			DMC		
		Pressure (mmHg)			Pressure (mmHg)		
		760	880	940	760	880	940
**Pressure (mmHg)**	760	/	0.92	1.54	/	0.28	0.48
	880	0.92	/	0.85	0.28	/	0.21
	940	1.54	0.85	/	0.48	0.21	/

Note: Effect size analyses for the Angle θ between α1N and α1C of the α1-helix in PD domain and the DMC between the tip of β3β4-hairpin of PD domain and its interacting loop from the RBM motif across different pressure cohorts.

Table S7. Effect size analyses for the Rgyr, DMC, and Angle α presented in Figure 5.

**Cohen's d**		Rgyr			DMC			Angle α		
		Pressure (mmHg)			Pressure (mmHg)			Pressure (mmHg)		
		760	880	940	760	880	940	760	880	940
**Pressure (mmHg)**	760	/	-0.12	0.11	/	-0.26	-0.02	/	-0.33	0.01
	880	-0.12	/	0.23	-0.26	/	0.25	-0.33	/	0.37
	940	0.11	0.23	/	-0.02	0.25	/	0.01	0.37	/

Note: Effect size analyses for the Rgyr of the binding interface of the complex, DMC between the RBM motif and the interaction surface of the PD domain, and cross angle α of the outer surface of the RBM motif across different pressure cohorts.

Table S8. Effect size analyses for the Angle θ and DMC presented in Figure 8.

Cohen's d		Angle θ				DMC			
		Temperature (K)				Temperature (K)			
		305	310	315	350	305	310	315	350
Temperature (K)	305	/	-0.18	/	/	/	0.03	/	/
	310	-0.18	/	-0.31	-0.16	0.03	/	-0.19	-0.53
	315	/	-0.31	/	0.13	/	-0.19	/	-0.42
	350	/	-0.16	0.13	/	/	-0.53	-0.42	/

Note: Effect size analyses for the Angle θ between α1N and α1C of the α1-helix in PD domain and DMC between the tip of β3β4-hairpin of PD domain and its interacting loop from the RBM motif across different temperature cohorts.

Table S9. Effect size analyses for the Rgyr, DMC, and Angle α presented in Figure 9.

Cohen's d		Rgyr				DMC				Angle α			
		Temperature (K)				Temperature (K)				Temperature (K)			
		305	310	315	350	305	310	315	350	305	310	315	350
Temperature (K)	305	/	0.15	/	/	/	-0.45	/	/	/	-0.46	/	/
	310	0.15	/	0.23	-0.60	-0.45	/	0.47	-0.51	-0.46	/	-0.24	-0.54
	315	/	0.23	/	-0.73	/	0.47	/	-0.74	/	-0.24	/	-0.30
	350	/	-0.60	-0.73	/	/	-0.51	-0.74	/	/	-0.54	-0.30	/

Note: Effect size analyses for the Rgyr of the binding interface of the complex, DMC between the RBM motif and the interaction surface of the PD domain and Cross angle α of the outer surface of the RBM motif across different temperature cohorts.

Table S10. Thermodynamic evaluation of the interaction between RBD and hACE2-PD under different pressures and temperatures.

Group	ΔG (kcal/mol)	ΔH (kcal/mol)	TΔS (kcal/mol)
760 mmHg/310 K	-83.57±0.85	-1488.38	-1404.81
880 mmHg/310 K	-96.47±0.98	-1479.5	-1383.03
940 mmHg/310 K	-121.48±0.82	-1384.44	-1262.96
880 mmHg/305 K	-117.91±0.85	-1544.41	-1426.5
880 mmHg/315 K	-37.35±0.76	-1447.06	-1409.71
880 mmHg/350 K	-38.88±1.05	-1405.31	-1366.43

Note: ΔG was calculated using Delphi. TΔS were calculated using MolAICal software. ΔH was calculated as ΔH=TΔS+ΔG.

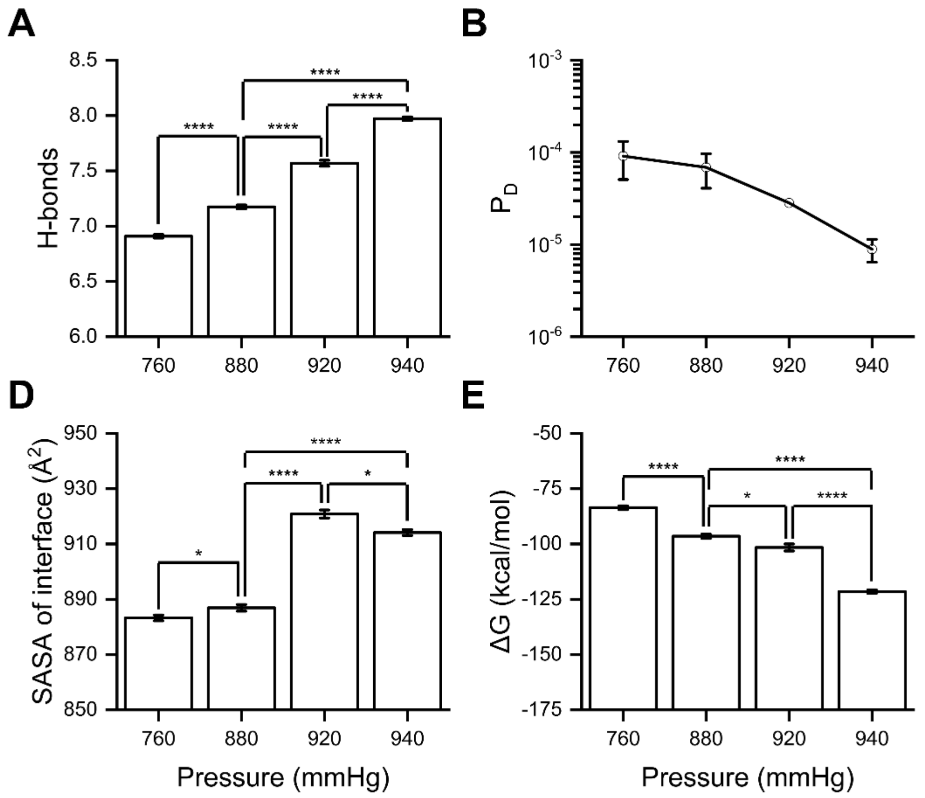

Figure S1. High pressure enhances RBD/PD binding. The average (A) number of H-bonds, (B) dissociation probability (PD), (C) SASA of the interface, and (D) binding energy (ΔG, MM-PBSA) under different pressures (760, 880, 920, and 940 mmHg) at a constant temperature of 310 K. Data are shown as mean ± SEM. Statistical significance was analyzed using one-way ANOVA followed by Tukey's multiple comparisons test. **** indicates $p < 0.0001$, * indicates $p < 0.05$, and NS indicates not significant. The data for 920 mmHg were extracted from a single 100 ns trajectory, rather than from three replicate experiments.
